# Supplementary figures and images for: The Six-Transmembrane Enzyme GDE2 Is Required for the Release of Molecularly Distinct Small Extracellular Vesicles from Neurons
Source: Cells. 2024 Aug 24;13(17):1414. doi: 10.3390/cells13171414 (PMC11394063; doi:10.3390/cells13171414)

**$\alpha$ -FLAG**  
(GDE2)

**Vector**

**GDE2**

**GDE2-H243A**

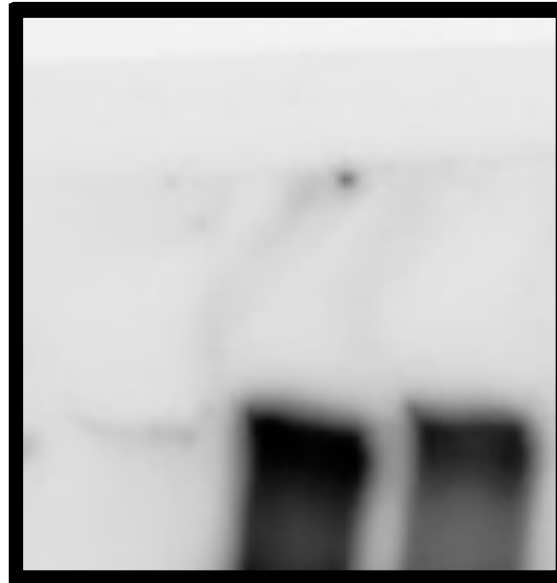

-100 kDa

-70 kDa

Supplement: Supplementary file 1 [file cells-13-01414-s001.zip › Figure S1.pdf]

A

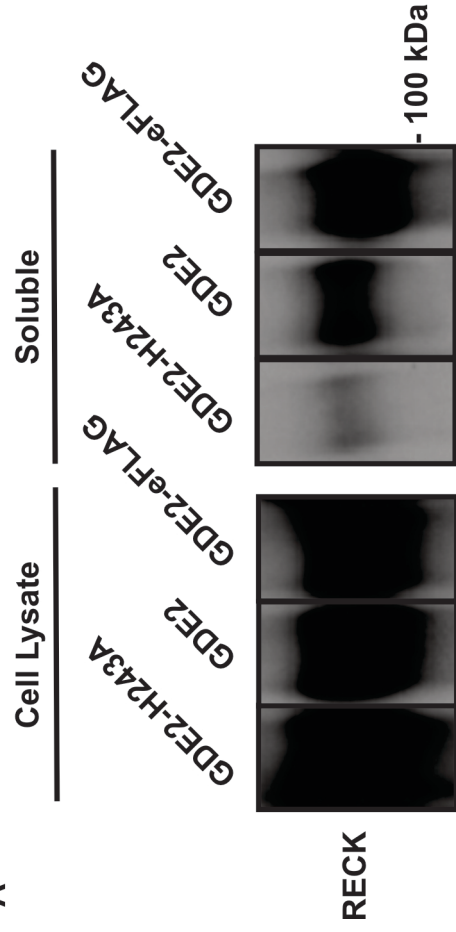

B

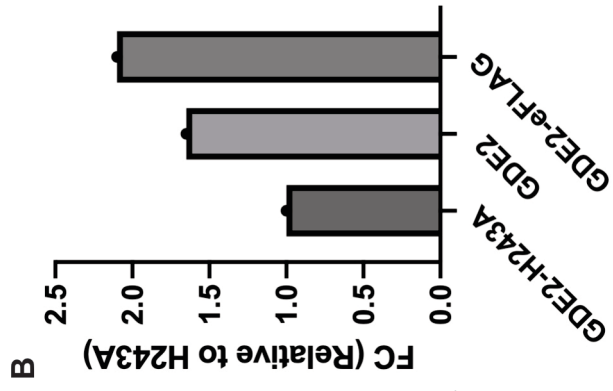

C

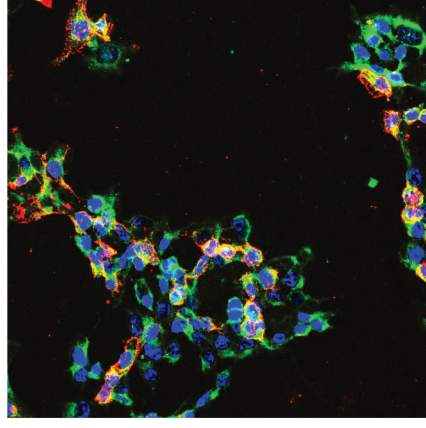

Supplement: Supplementary file 1 [file cells-13-01414-s001.zip › Figure S2.pdf]
